# Chromosomal copy number variations in products of conception from spontaneous abortion by next-generation sequencing technology

**DOI:** 10.1097/MD.0000000000018041

**Published:** 2019-11-22

**Authors:** Rulin Dai, Qi Xi, Ruixue Wang, Hongguo Zhang, Yuting Jiang, Leilei Li, Ruizhi Liu

**Affiliations:** Center of Reproductive Medicine and Center of Prenatal Diagnosis, the First Hospital, Jilin University, Changchun, Jilin, China.

**Keywords:** fetal chromosomal abnormality, next-generation sequencing, product of conception, spontaneous miscarriage

## Abstract

Fetal chromosomal abnormalities are considered to be the main cause of spontaneous abortion (SA). We aimed to determine the differences in the rates and numbers of chromosomal abnormalities between samples from women with a history of one versus more than one SA as well as between samples from first- and second-trimester SAs in women from Northeast China.

In total, 1210 products of conception (POCs) from patients with a history of one or more SAs were examined. Of these 1210 samples, 434 were from women with a history of 1 SA, and 776 were from women with a history of more than 1 SA. Additionally, 1071 samples were from the first trimester, 118 were from the second trimester, and 21 were from the third trimester. We identified chromosomal abnormalities by next-generation sequencing (NGS) technology. Among the 1210 POCs in women with SA, 607 (50.17%) had fetal chromosomal abnormalities. There were no significant differences in the rates of chromosomal abnormalities according to the abortion frequency. However, first-trimester SA had a significantly higher percentage of fetal chromosomal abnormalities than second-trimester SA (*P* < .05). Among 663 chromosomal abnormalities, 633 abnormalities occurred in first-trimester SA; the most frequent karyotype was trisomy 16 (14.38%), followed by monosomy X (13.27%), trisomy 22 (7.90%), and trisomy 15 (5.37%). Thirty abnormalities occurred in second-trimester SA; the most frequent karyotype was trisomy 18 (26.67%), followed by monosomy X (16.67%), trisomy 21 (13.33%), and trisomy 13 (10.00%). No chromosomal abnormalities occurred in the third trimester.

These findings indicate the importance of determining the genetic cause of abortion in patients with a history of SA. We also identified a trend suggesting that the percentage of fetal chromosomal abnormalities is significantly higher in first- than second-trimester SA. The detection rate of chromosomal abnormalities in POCs from SA can be increased by NGS, which is beneficial for couples with recurrent miscarriages and offers better genetic counseling in the clinical setting.

## 1 Introduction

Spontaneous abortion (SA) is defined as the spontaneous loss of a clinically established intrauterine pregnancy before the fetus has reached viability. About 15% to 20% of clinical pregnancies result in spontaneous miscarriage, and about 25% of all women experience at least one abortion.^[1–3]^ Recurrent miscarriage is classically defined as three or more consecutive miscarriages.^[4,5]^ However, many researchers have now revised this term to recurrent pregnancy loss, which is defined as two or more pregnancy losses, because of the recent increase in the prevalence of childless couples. The estimated incidences of recurrent miscarriage and recurrent pregnancy loss are 1% and 5%, respectively.^[4,5]^ Numerous studies have evaluated the relationships between SA and genetic, endocrinological, anatomical, infectious, and autoimmune factors. Our laboratory historically had studied and determined the relationship between fetal chromosomal abnormalities and maternal age during first trimester SA, and we found that the kinds of fetal abnormalities, numbers of abortions, and chromosomal abnormality rates increased with increasing maternal age.^[3]^

Fetal chromosomal aneuploidies are the main etiology of SA,^[6]^ with aneuploidy and polyploidy of chromosomes 13, 16, 18, 21, 22, X, and Y being particularly frequent.^[7,8]^ G-banding karyotyping of routine chromosome analysis has been the gold standard for cytogenetic diagnosis.^[9]^ Within the last 10 years, chromosomal high-throughput genetic technology has been increasingly adopted to detect submicroscopic pathogenic copy number variations (CNVs) in genetic diagnoses.^[10,11]^

In the present study, we examined products of conception (POCs) of SAs to identify chromosomal abnormalities using next-generation sequencing (NGS) technology. We also investigated differences in the rates and numbers of chromosomal abnormalities in samples from women with a history of one SA versus two or more SAs, as well as in samples from first- vs second-trimester SAs in women from Northeast China.

## 2 Subjects and methods

### 2.1 Subjects and study design

We evaluated 1210 POCs from women who had a history of one or more SAs, had no children, and attended the outpatient abortion clinic of the Reproductive Medicine Department of the First Hospital of Changchun, Jilin Province, Northeastern China from 15 October 2016 to 26 March 2019.

We excluded samples from women with SA if either the woman or her husband had chromosome abnormalities; if the woman had structural abnormalities of the genital organs; and if the woman had major diseases such as diabetes or thyroid hypofunction. Pregnancies conceived in women with a history of either one SA (434 cases) or more than one SA (776 cases) were included, including SAs during the first trimester (1071 cases), second trimester (118 cases), and third trimester (21 cases).

This study was approved by the ethics committee of the First Hospital of Changchun, Jilin Province (No. 2016–432), and all patients provided informed consent to participate in the study.

### 2.2 Chromosomal CNVs by NGS and validation

Genomic DNA was isolated from chorionic villi or tissue using a TIANamp Genomic DNA Kit (Beijing Tiangen Biotech Co. Ltd., Beijing, China). Genomic DNA from POCs was sheared to 250 to 300 bp fragments using an Ion Shear Plus Reagents Kit (Thermo Fisher Scientific, Waltham, MA). Ion Torrent barcoded libraries were made using an Ion Plus Fragment Library Kit (Thermo Fisher Scientific). An Ion PGM Template OT2 200 Kit (Thermo Fisher Scientific) was used for template amplification and enrichment of the target sequence. Ion sphere particles were recovered, and template-positive ion sphere particles were enriched using an Ion OneTouch ES (Thermo Fisher Scientific). Sequencing was performed using an Ion PGM Sequencing 200 Kit v2 (Thermo Fisher Scientific) on a 318 sequencing chip for a total of 500 nucleotide flows, yielding average read lengths of 220 to 230 bp. Ten DNA samples were pooled together and labeled with different barcodes on the 318 chip. The average whole genomic sequence depth was approximately 0.02×, and the average read number was approximately 500 K. The primary sequencing BAM data were submitted to the PGX cloud server (available at http://www.pgxcloud.com/), which was offered by a third-party company (JBRH, China), to analyze the chromosomal CNVs. The data analysis pipeline was established according to previous reports.^[12,13]^

### 2.3 Karyotype analysis

Peripheral blood samples were collected in sterile tubes containing 30 IU/ml heparin and aseptically inoculated into lymphocyte culture solution (Yishengjun; Guangzhou Baidi Biotech, Guangzhou, China). Cultures were incubated at 37°C for 72 hours and then treated with 20 μg/ml of colcemid for 1 hour. G-banding of metaphase chromosomes was performed by standard methods.^[14]^ A minimum of 30 metaphase cells were counted for each individual, and at least 5 cells were analyzed. Chromosome abnormalities were described according to the criteria established by the International System for Human Cytogenetic Nomenclature.^[14]^

### 2.4 Statistical analysis

The data were compared using Student *t* test or one-way analysis of variance, as appropriate, and statistically analyzed using SPSS software ver. 11.5 (SPSS, Inc., Chicago, IL). Differences were considered statistically significant when *P* < .05.

## 3 Results

Among 1210 chorionic villi or tissue samples of SAs, 434 samples were obtained from women with a history of 1 SA, and 776 samples were obtained from women with a history of more than 1 SA. Additionally, 1071 samples were from the first trimester, 118 were from the second trimester, and 21 were from the third trimester. All 1210 couples had normal chromosomes.

**Figure 1:**
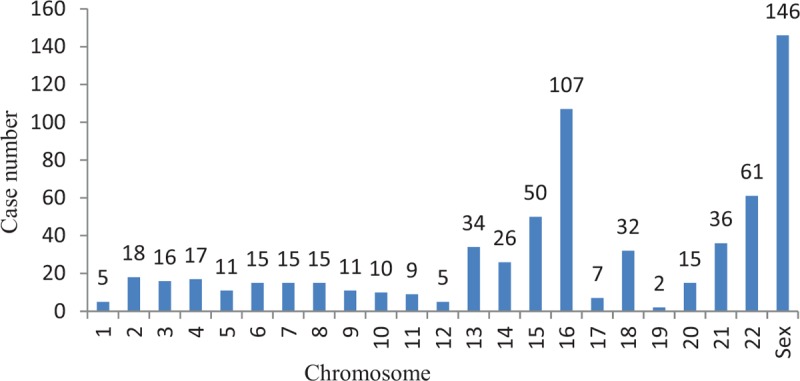
Distribution of chromosomal abnormalities in 1210 products of conception.

Among all 1210 samples, 603 (49.83%) had normal fetal chromosomes and 607 (50.17%) had fetal chromosomal abnormalities. Among the 607 samples with fetal chromosomal abnormalities, 212 (34.93%) were obtained from women with a history of one SA and 395 (65.07%) were obtained from women with a history of more than one SA. Additionally, among the 607 samples with fetal chromosomal abnormalities, 578 (95.22%) were from the first trimester and 29 (4.78%) were from the second trimester; no fetal chromosomal abnormalities were found in samples from the third trimester. There were no significant differences in the rates of chromosomal abnormalities according to the abortion frequency (*P* > .05) (Table 1). However, the first-trimester SAs had a significantly higher percentage of fetal chromosomal abnormalities than the second-trimester SAs (*P* < .05) (Table 2). Because some abnormalities involved two or more chromosomes, the 607 samples contained 663 abnormalities. The autosomal abnormalities of the 607 samples included 6 kinds of abnormalities; the most frequent was trisomy (56.11%, 372/663), followed by abnormalities involving 2 or more chromosomes (15.99%, 106/663) (Table 3, Fig. 1).

**Table 1:**
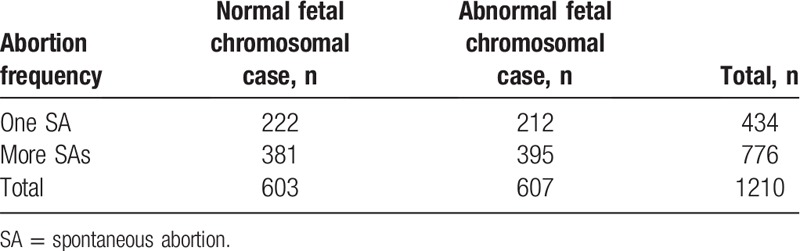
Fetal chromosomal abnormalities according to the abortion frequency.

**Table 2:**
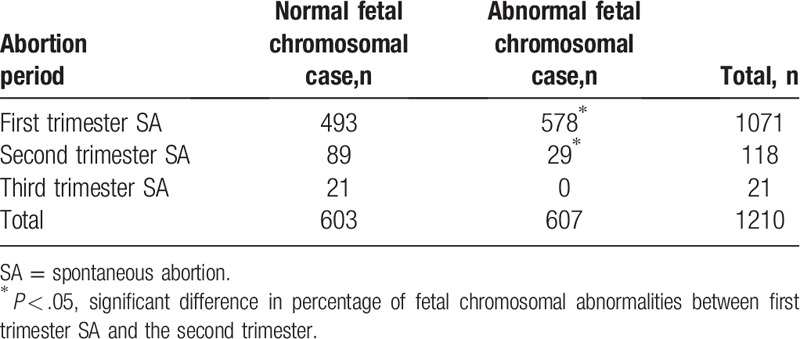
Fetal chromosomal abnormalities in the period of SAs.

**Table 3:**
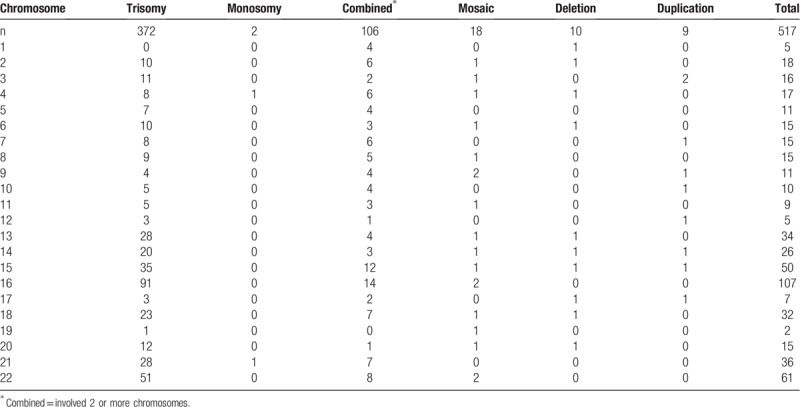
Autosome abnormalities in 1210 products of conception.

The abnormalities observed in this study involved all chromosomes, especially chromosome 16, followed by chromosomes X and 22. However, autosomal abnormalities were rare on chromosomes 19, 1, and 12 (Tables 3 and 4, Fig. 1). The 517 autosome chromosome abnormalities included 10 (1.93%) related to deletion and 9 (1.74%) related to duplication (Table 5).

**Table 4:**
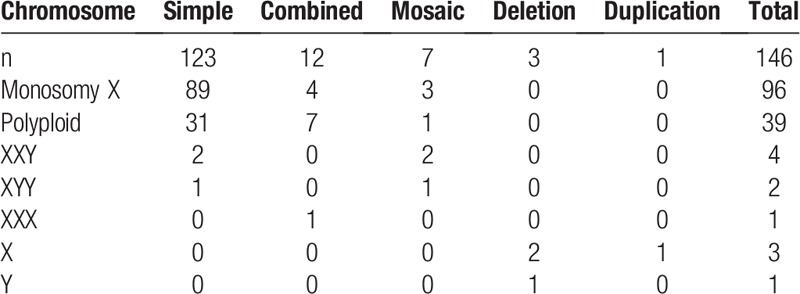
Sex chromosomal abnormalities in products of conception.

**Table 5:**
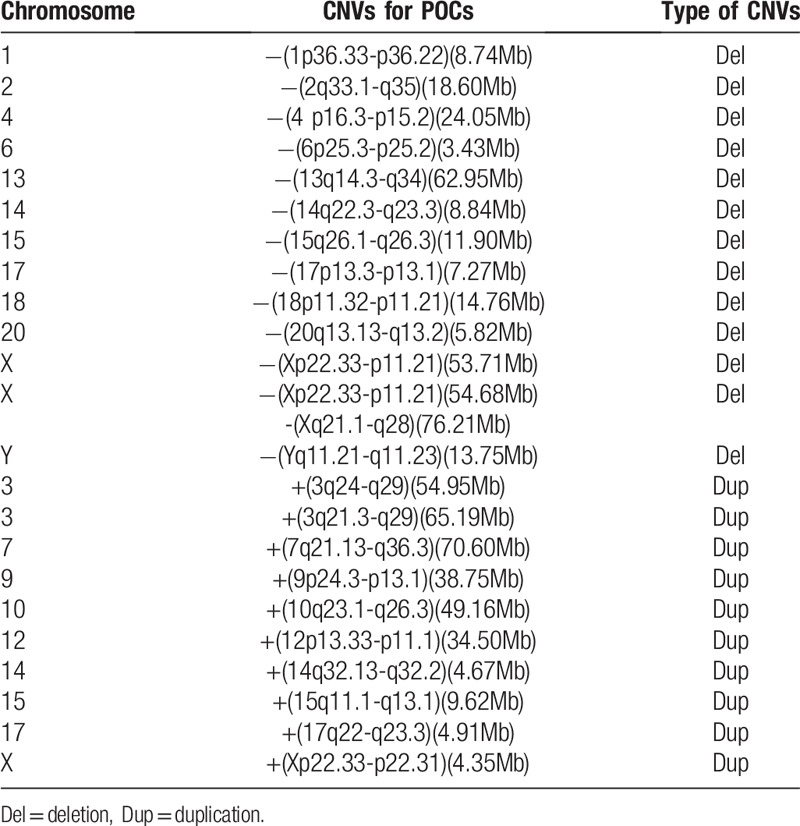
Deletion/ duplication abnormalities in products of conception.

In total, 146 of the 607 samples had sex chromosomal abnormalities, accounting for 22.02% (146/663) of all abnormalities. The 146 sex chromosome abnormalities included 96 (65.75%) related to monosomy X, 39 (26.71%) related to polyploidy and 4 (2.74%) related to deletion/duplication (Table 5).

**Figure 2:**
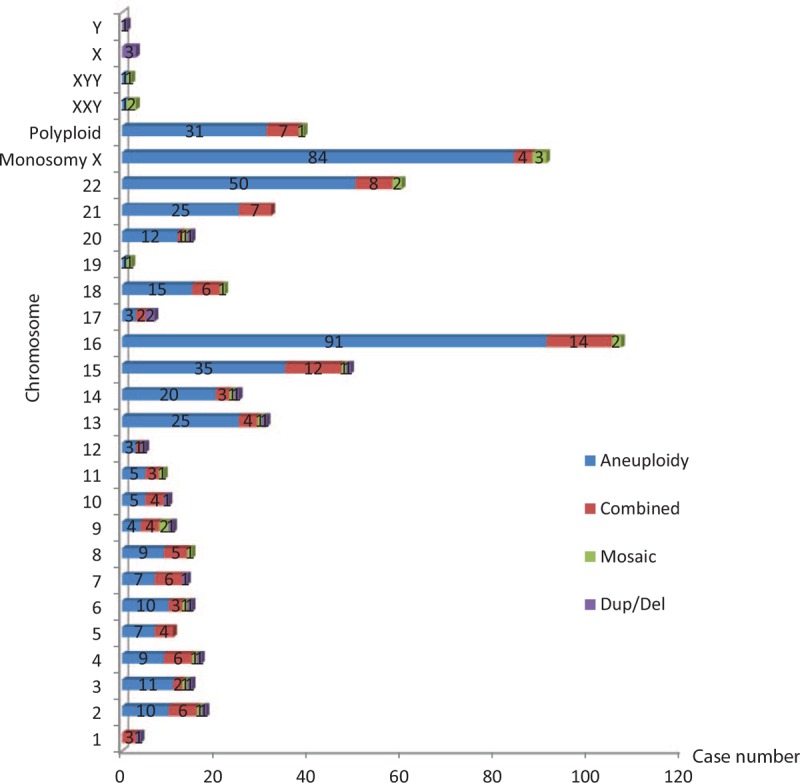
Distribution of chromosomal abnormalities in the first trimester. Del = deletion, Dup = duplication.

**Figure 3:**
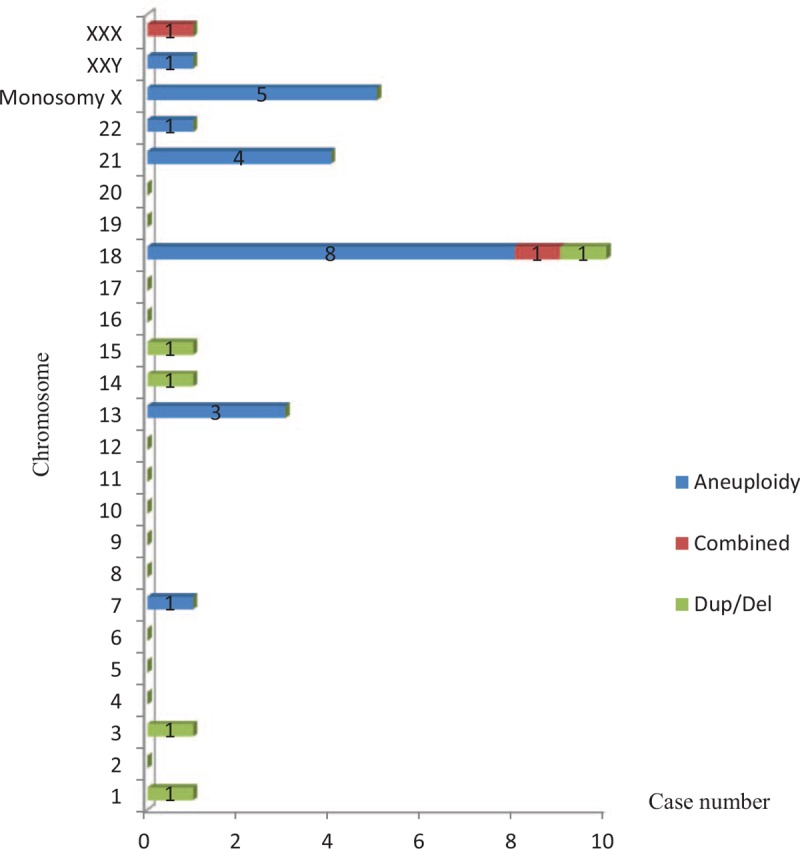
Distribution of chromosomal abnormalities in the second trimester. Del = deletion, Dup = duplication.

In total, 633 of the 663 chromosomal abnormalities occurred in first-trimester SAs, and these abnormalities mainly involved chromosomes 16, sex, 22, and 15. The most frequent karyotype was trisomy 16 (14.38%, 91/633), followed by monosomy X (13.27%, 84/633), trisomy 22 (7.90%, 50/633), and trisomy 15 (5.37%, 34/633) (Fig. 2). Thirty abnormalities occurred in second-trimester SAs, and these abnormalities mainly involved chromosomes 18, sex, 21, and 13. The most frequent karyotype was trisomy 18 (26.67%, 8/30), followed by monosomy X (16.67%, 5/30), trisomy 21 (13.33%, 4/30), and trisomy 13 (10.00%, 3/30) (Fig. 3).

## 4 Discussion

Many factors may contribute to SA, including genetic, endocrinological, and anatomical factors as well as infectious, autoimmune, and systemic maternal diseases.^[15,16]^ However, chromosomal abnormalities have long been recognized as the major cause of SA, with numerical chromosome abnormalities accounting for 50% to 78% of all SAs.^[17,18]^ In the current study, we investigated differences in the rates and numbers of chromosomal abnormalities between samples obtained from women with a history of one SA and 2 or more SAs as well as between samples from first- and second-trimester SAs in women from Northeast China.

We detected an overall chromosomal abnormality rate of 50.17% among 1210 POCs using high-throughput genetic technology in Northeast China. Chromosomal abnormalities were detected in all chromosomes. A total of 776 samples were obtained from women with a history of more than one SA, but there were no significant differences in the rates of chromosomal abnormalities according to the abortion frequency. Recent studies have suggested that aneuploidy rates decrease with the number of prior miscarriages.^[19,20]^ Our results suggest that in patients with a history of SA, examination of POCs from the SAs to determine the genetic cause is essential. If no genetic factors of POCs are identified, women with SA may uncover unrelated causes of abortion, leading to unnecessary treatment.

However, patients in the first trimester had a significantly higher percentage of chromosomal abnormalities than those in the second trimester, and no chromosomal abnormalities of POCs were found in the third trimester. Further research is needed because of the limited number of samples in this study. We speculate that the cause of abortion in the third trimester has little to do with fetal chromosomal abnormalities, and other tests are needed to identify the cause. The abnormalities of different chromosomes corresponded to SAs in different trimesters. Chromosomal abnormalities in samples from first-trimester SAs were detected in all chromosomes. The abnormalities mainly involved chromosome 16; the most frequent karyotype was trisomy 16. The most frequent karyotype of second-trimester SAs was trisomy 18, and the abnormalities in the second trimester occurred on 11 chromosomes.

Cytogenetic studies have shown that most abnormalities are numerical chromosome abnormalities (86%), with a minority caused by chromosome mosaicism (8%) and structural chromosome abnormalities (6%).^[5]^ Conventional karyotyping is currently considered the gold standard of detecting the chromosome karyotype. Additionally, cytogenetics can detect low-level mosaicism below the threshold detected by molecular methods.^[21]^ Due to the band resolution of this method, submicroscopic deletions and duplications cannot be detected less than 5Mb typically, but NGS technology could achieve. In our study, we found that chromosome mosaicism of POCs accounted for 3.71% (25/663) of all chromosomal abnormalities detected by high-throughput genetic technology.

Traditional detection method has been unable to meet the demand of all-sided detection. Fluorescence in situ hybridization (FISH) analysis of chorionic villi was performed on chromosomes 13, 16, 18, 21, 22, X, and Y with aneuploidy and polyploidy. We found that the abnormalities detected by FISH technology accounted for only 61.69% (409/663) of all chromosomal abnormalities detected by high-throughput genetic technology. If FISH technology is used for detection, some abnormalities will be missed. Our laboratory historically used FISH technology for testing, and An^[8]^ found that among patients with recurrent abortions, abortus aneuploidy occurred more frequently than sporadic miscarriages (40.54% vs 33.64%, respectively). The rate of abnormalities detected by high-throughput genetic technology in the present study was higher than that found in the study by An.^[8]^ We also found that detecting chromosomal CNVs by NGS could reduce the rate of omission.

For women, miscarriage is an unanticipated, physically and emotionally traumatic burden. Because many miscarriages have no clear medical cause, a sense of guilt and self-accusation is often prominent.^[20,22]^ Therefore, for many women, the absence of POC results means that the most important factor related to SA will be ignored and findings unrelated to the SA will be uncovered, increasing the economic and psychological burden and resulting in unnecessary treatment.

## 5 Conclusion

Our findings highlight the importance of determining the genetic cause of abortion in patients with a history of SA. We also identified a trend suggesting that the percentage of fetal chromosomal abnormalities in first-trimester SA is significantly higher than that in second-trimester SA. The detection rate of chromosomal abnormalities in POCs from SA can be increased by next-generation sequencing, which is beneficial for couples with recurrent miscarriages and offers better genetic counseling in the clinical setting.

## Author contributions

**Conceptualization:** Ruizhi Liu.

**Data curation:** Qi Xi.

**Funding acquisition:** Rulin Dai, Ruizhi Liu.

**Investigation:** Qi Xi, Ruixue Wang.

**Methodology:** Hongguo Zhang, Leilei Li.

**Software:** Yuting Jiang.

**Writing – original draft:** Rulin Dai.

**Writing – review & editing:** Ruizhi Liu.
